# Pathology-validated prevalence and clinical characteristics of coronary medial arterial calcification

**DOI:** 10.1093/ehjopen/oeag076

**Published:** 2026-05-15

**Authors:** Yuki Matsumoto, Sho Torii, Kazuki Aihara, Yuto Ono, Yu Sato, Norihito Nakamura, Manabu Shiozaki, Tsukasa Kato, Masataka Nakano, Tomoya Onodera, Yuki Egawa, Shingo Ito, Kaho Hashimoto, Ryosuke Omura, Daiki Suzuki, Yohei Masugi, Yoshihiro Morino, Yuji Ikari, Gaku Nakazawa

**Affiliations:** Department of Cardiology, Tokai University School of Medicine, 143 Shimokasuya, Isehara, Kanagawa 259-1193, Japan; Department of Cardiology, Tokai University School of Medicine, 143 Shimokasuya, Isehara, Kanagawa 259-1193, Japan; Department of Cardiology, Tokai University School of Medicine, 143 Shimokasuya, Isehara, Kanagawa 259-1193, Japan; Department of Cardiology, Tokai University School of Medicine, 143 Shimokasuya, Isehara, Kanagawa 259-1193, Japan; Department of Cardiology, Tokai University School of Medicine, 143 Shimokasuya, Isehara, Kanagawa 259-1193, Japan; Department of Cardiology, Tokai University School of Medicine, 143 Shimokasuya, Isehara, Kanagawa 259-1193, Japan; Department of Cardiology, Tokai University School of Medicine, 143 Shimokasuya, Isehara, Kanagawa 259-1193, Japan; Department of Cardiology, Akita University School of Medicine, 1-1-1 Hondo, Akita, Akita 010-8543, Japan; Department of Cardiology, Ageo Central General Hospital, 1-10-10 Kashiwaza, Ageo, Saitama 362-8588, Japan; Department of Cardiology, Shizuoka City Shizuoka Hospital, 10-93 Ote-machi, Aoi-ku, Shizuoka, 420-8630, Japan; Department of Cardiology, Shizuoka City Shizuoka Hospital, 10-93 Ote-machi, Aoi-ku, Shizuoka, 420-8630, Japan; Department of Cardiovascular Medicine, St.Marianna University Hospital, 2-16-1 Sugao, Miyamae-ku, Kawasaki, Kanagawa 216-8511, Japan; Department of Cardiology, Tokai University School of Medicine, 143 Shimokasuya, Isehara, Kanagawa 259-1193, Japan; Department of Cardiology, Tokai University School of Medicine, 143 Shimokasuya, Isehara, Kanagawa 259-1193, Japan; Department of Cardiology, Tokai University School of Medicine, 143 Shimokasuya, Isehara, Kanagawa 259-1193, Japan; Department of Pathology, Tokai University School of Medicine, 143 Shimokasuya, Isehara, Kanagawa 259-1193, Japan; Division of Cardiology, Department of Internal Medicine, Iwate Medical University, 2-1-1 Idaidori, Yahaba-cho, Shiwa-gun, Iwate 028-3694, Japan; Department of Cardiology, Tokai University School of Medicine, 143 Shimokasuya, Isehara, Kanagawa 259-1193, Japan; Department of Cardiology, Kindai University School of Medicine, 377-2 Ohno-Higashi, Osaka-Sayama, Osaka, Japan

## Abstract

**Aims:**

Medial arterial calcification (MAC) is well characterized in peripheral arteries, but its pathology-validated prevalence in the coronary circulation remains uncertain. Because intravascular imaging cannot routinely delineate the elastic laminae in advanced atherosclerotic lesions, deep intimal calcification (DIC) accumulating adjacent to the internal elastic lamina (IEL) may fundamentally mimic MAC. This study aimed to determine the strict pathology-validated prevalence of true coronary MAC and morphologically characterize DIC as a potential imaging mimic using a large-scale autopsy registry.

**Methods and results:**

We evaluated 4508 histologic sections from 327 coronary arteries in 112 autopsy cases. To ensure accurate layer-specific differentiation, the IEL and external elastic lamina were precisely identified using Movat Pentachrome and EVG stains. The cohort was high-risk, featuring 85% cardiac deaths. Pathology-confirmed MAC was remarkably rare, present in only 1.0% of sections and 3.6% of patients. Strikingly, coronary MAC presented in two distinct morphological phenotypes. Three patients exhibited nodular MAC strictly co-existing with intimal nodules in severely stenotic segments, representing secondary mechanical protrusion from the intima. Conversely, only one patient exhibited isolated medial sheet calcification with minimal intimal narrowing, representing true primary medial disease. Notably, the IEL-overriding pattern occasionally seen in peripheral MAC was entirely absent. Meanwhile, DIC was significantly more prevalent, identified in 35.7% of patients.

**Conclusion:**

True primary coronary MAC is exceptionally rare. Because dense acoustic shadowing clinically conceals secondary medial nodular protrusions *in vivo*, imaging-reported coronary ‘MAC’ almost exclusively represents DIC. Recognizing three distinct phenotypes—DIC, secondary nodular protrusion, and the rare true Mönckeberg-type MAC—is essential to accurately interpret clinical imaging findings.

## Graphical Abstract

**Graphical Abstract:**
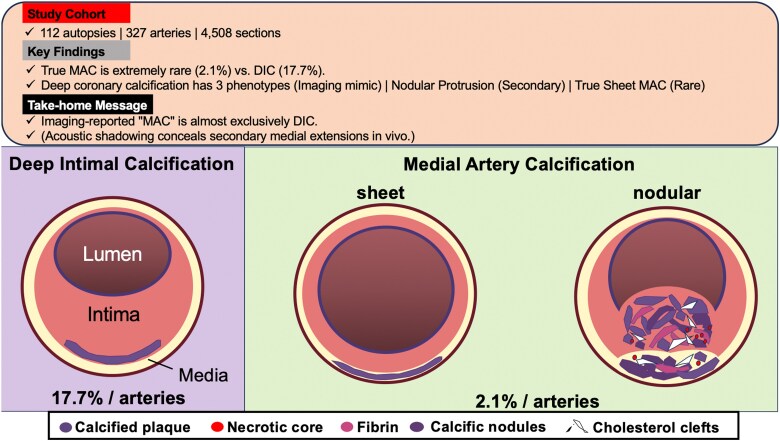

**Editorial for this article: Eur Heart J Open 2026; https://doi.org/10.1093/ehjopen/oeag093.**

## Introduction

Arterial calcification occurs in two principal layers: intimal calcification within atherosclerotic plaque and medial arterial calcification (MAC) within the tunica media.^1^ While MAC is a well-established pathology in the lower extremities, its status in the coronary circulation remains controversial. Although intravascular imaging studies have reported coronary ‘MAC’ in 7–18% of lesions,^2^ visualizing the internal elastic lamina (IEL) is often difficult especially in advanced atherosclerotic lesions. Consequently, it remains unknown whether the imaging signature of ‘coronary MAC’ represents true medial pathology or a different calcific phenotype.

We hypothesized that deep intimal calcification (DIC), defined as intimal calcium accumulated immediately adjacent to the IEL, may fundamentally mimic the appearance of MAC in clinical imaging. Furthermore, we sought to clarify the morphological relationship between true coronary MAC and intimal atherosclerosis, to determine whether it represents an independent Mönckeberg-type sclerosis—as seen in peripheral vessels—or a distinct phenotype.^3^ Therefore, the primary aim of this study was to estimate the strict pathology-validated prevalence of true coronary MAC using a large-scale multicentre autopsy registry.

## Methods

We analysed 327 coronary arteries from 112 autopsy cases (4508 sections) from our registry.^4^ The methodology and scientific validity of this registry have been previously described in detail.^4^ Briefly, hearts were perfusion-fixed in 10% buffered formalin under physiologic pressure and radiographed; epicardial arteries were then removed and re-radiographed. Severely calcified lesions underwent ethylenediaminetetraacetic acid decalcification, and the arteries were sequentially segmented at 3–4 mm intervals. To achieve precise identification of the IEL and EEL, all 4508 sections were evaluated using high-resolution slides with Movat Pentachrome and/or EVG stains. This dual-staining approach was essential for verifying elastic fibre continuity by referencing healthy adjacent segments, a critical step in differentiating the tunica media from the deep intima in advanced atherosclerotic lesions where the IEL is often fragmented. Morphometric analysis was conducted using image-analysis software (Zen2, Carl Zeiss), and findings were corroborated at the section, artery, and patient levels. DIC was defined as intimal calcium located immediately adjacent to the IEL, occupying less than 20% of the total intimal thickness. Atherosclerotic plaques were classified according to established criteria. The study protocol was approved by the Institutional Review Board (25R132).

## Results

The mean age of the cohort was 73.0 ± 11.8 years, with a male predominance (78%). Haemodialysis was present in 21.4% of patients. Baseline clinical data were complete for all 112 patients, while laboratory values were available for 100 patients (89%). Notably, the cohort was characterized by a high prevalence of cardiac deaths, which occurred in 95 of the 112 patients (85%). Among these, coronary artery disease was the primary cause of death in 51 patients (46%) (*Table 1*). Advanced intimal atherosclerosis (fibrocalcific or fibroatheroma) was identified in 66.5% of all analysed sections (*Table 1*).

**Table 1:** Patient, lesion, and section characteristics

Patient characteristics	(*n* = 112)	Cause of death	(*n* = 112)
Age, years	73.0 ± 11.8	Cardiac death, *n* (%)	95 (84.8)
Male sex, *n* (%)	87 (77.7)	Non-cardiac death, *n* (%)	17 (15.2)
BH, m	1.61 ± 0.08	Lesion characteristics	(*n* = 327)
BW, kg	59.6 ± 12.3	Right coronary artery, *n* (%)	87 (26.6)
BMI, kg/m^2^	22.9 ± 4.2	Left main trunk, *n* (%)	54 (16.5)
BSA, m2	1.62 ± 0.2	Left anterior descending artery, *n* (%)	100 (30.6)
Comorbidities	(*n* = 112)	Left circumflex artery, *n* (%)	86 (26.3)
Diabetes mellitus, *n* (%)	55 (49.1)	No. of arteries treated with PCI, *n* (%)	62 (19.0)
Hypertension, *n* (%)	75 (67.0)	No. of arteries treated with CABG, *n* (%)	12 (3.7)
Dyslipidaemia, *n* (%)	56 (50.0)	Plaque type	(*n* = 4508)
Chronic kidney disease, *n* (%)	66 (58.9)	Non-advanced atherosclerotic plaque	
Haemodialysis, *n* (%)	24 (21.4)	Adaptive intimal thickening, *n* (%)	611 (13.6)
Smoking, *n* (%)	52 (46.4)	Pathological intimal thickening, *n* (%)	901 (20.0)
Laboratory data^a^	(*n* = 100)	Fibrous plaque, *n* (%)	187 (4.1)
LDL-C, mg/dL	77.0 (50–105)	Advanced atherosclerotic plaque	
HDL-C, mg/dL	38.1 (30–50)	Fibrocalcific plaque, *n* (%)	2203 (48.9)
TG, mg/dL	83.5 (53–121)	Sheet calcification, *n* (%)	1628 (36.1)
HbA1c, %	6.1 (5.6–7.1)	Nodular calcification, *n* (%)	567 (12.6)
Serum creatinine, mg/dL	1.6 (1.0–3.2)	Calcified nodule, *n* (%)	8 (0.2)
eGFR, mL/min/1.73 m2	29.6 (14.0–51.4)	Fibroatheroma, *n* (%)	578 (12.8)
Serum calcium, mg/dL	8.6 (8.0–9.2)	Thin cap fibroatheroma, *n*(%)	19 (0.4)
Serum phosphorus, mg/dL	4.3 (3.4–6.5)	Ruptured plaque, *n* (%)	9 (0.2)

BH, body height; BW, body weight; BMI, body mass index; BSA, body surface area; LDL-C, low-density lipoprotein cholesterol; HDL-C, high-density lipoprotein cholesterol; TG, triglyceride; HbA1c, haemoglobin A1c; eGFR, estimated glomerular filtration rate; PCI, percutaneous coronary intervention; CABG, coronary artery bypass grafting.

^a^Laboratory values were available for 100 patients (89%).

**Figure 1:**
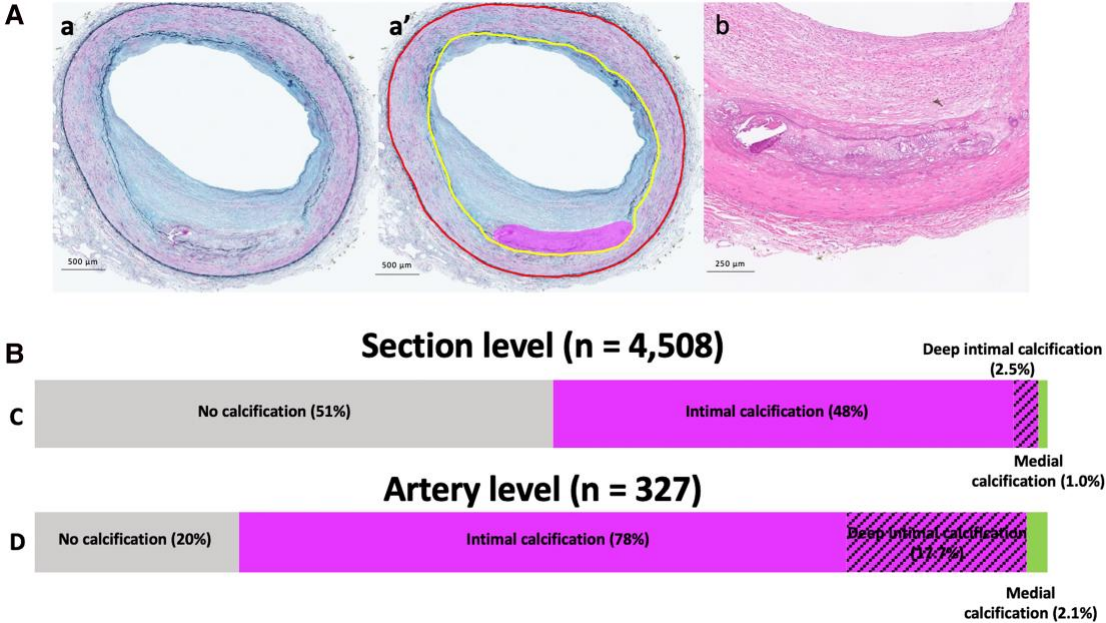
Histological characteristics and prevalence of DIC and MAC. (*A*) Deep intimal calcification (DIC). Movat Pentachrome (a, a′) and H&E (*B*) stains show intimal calcification adjacent to the internal elastic lamina (IEL). Schematics indicate IEL (yellow), external elastic lamina (EEL, red), and calcium (purple/green). (*C*, *D*) Prevalence at the section (*C*) and artery (*D*) levels. DIC (hatched purple) is significantly more prevalent than true MAC (green).

Pathology-confirmed MAC was remarkably rare, present in only 1.0% of sections (44/4508), 2.1% of arteries (7/327), and 3.6% of patients (4/112). All four MAC cases (mean age 68 years, three females) occurred exclusively in patients with diabetes and stage 5 CKD (two on haemodialysis). Strikingly, MAC presented in two distinct phenotypes. Three patients exhibited nodular MAC localized to the right coronary artery (RCA), diagonal branch, or left circumflex artery. In these cases, medial nodules co-existed exclusively with intimal nodules in severely stenotic segments (mean luminal stenosis: 95.0%), strongly suggesting secondary protrusion from the intima rather than primary medial disease. Conversely, only one patient exhibited extensive sheet MAC. Although accompanied by intimal nodules in the mid-segments of RCA, the sheet MAC extended widely into distal segments with minimal intimal narrowing (27.0% stenosis), representing the sole case of ‘true’ primary medial calcification (*Figure 1*).

**Figure 2:**
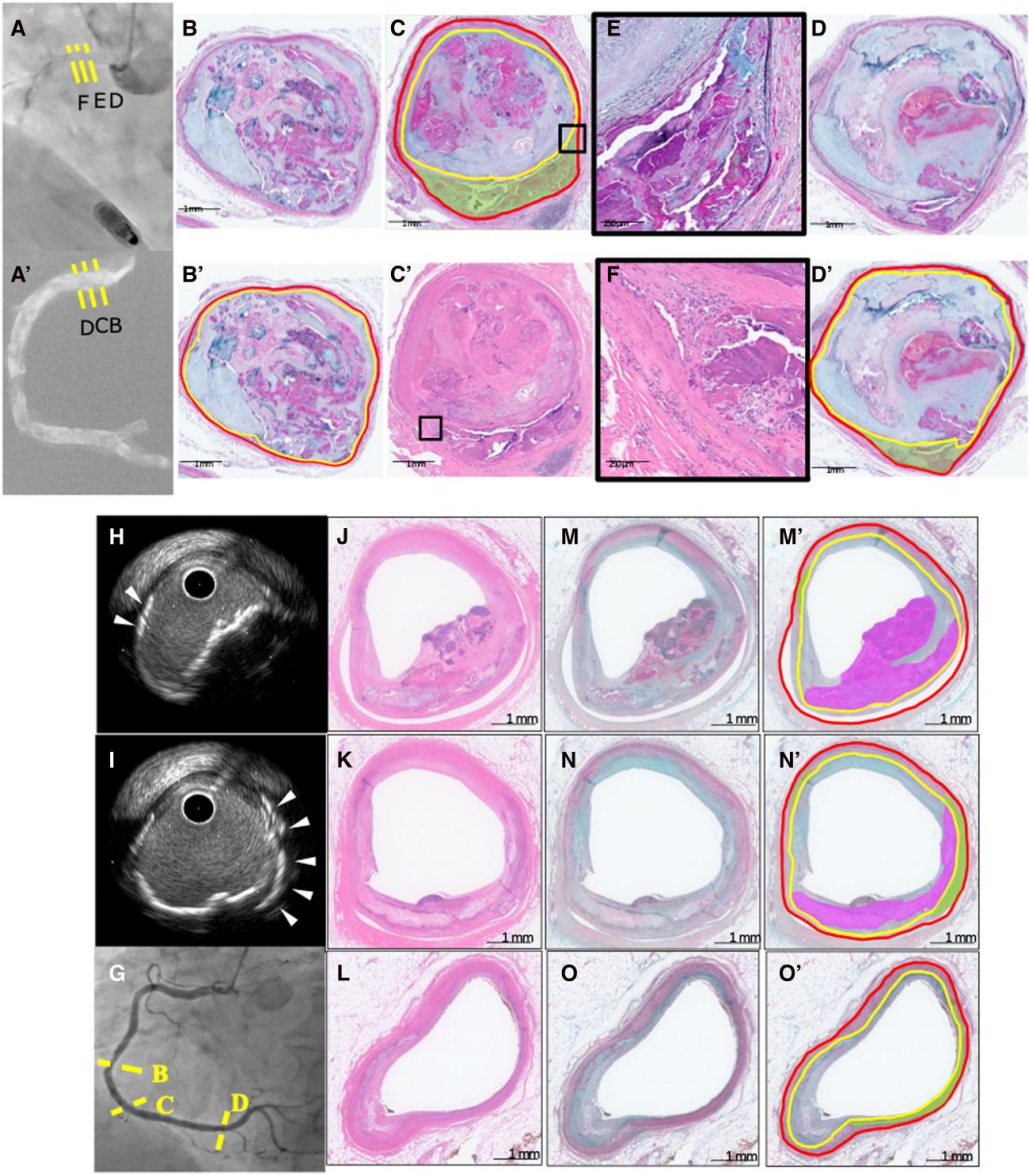
Angiographic, IVUS, and histological correlation of coronary MAC. (A–F) Secondary nodular protrusion. Angiography (*A*) and corresponding sections show intimal nodules (*B*) protruding into the media (*C*, *D*). (*G–O*) True medial sheet MAC and imaging limitations. Angiography (*G*) and IVUS (*H*, *I*) demonstrate circumferential calcification with dense acoustic shadowing. Corresponding histology (*J–N*) confirms medial sheet calcification. Crucially, acoustic shadowing on clinical imaging entirely conceals the underlying medial extension *in vivo*.

In distinct contrast to peripheral arteries where MAC in certain instances ‘overrides’ or focally extends just beyond the IEL, no such IEL-overriding patterns were identified in this coronary cohort; all identified MAC remained strictly confined to the medial layer (*Figure 2*).

On the other hand, DIC was identified in 35.7% of patients (40/112) and 17.7% of vessels (58/327), representing a substantially higher prevalence compared to MAC. Consistent with the findings for MAC, 100% of DIC lesions occurred exclusively within segments containing established atherosclerotic plaques; neither occurred in areas of isolated intimal hyperplasia.

## Discussion

Our study demonstrates that pathology-validated coronary MAC is an exceedingly rare phenomenon, found in only 3.6% of patients even in an ultra-high-risk population. Our data revealed that coronary medial calcification primarily manifests in two distinct morphological patterns. Three of our four MAC cases were nodular and strictly accompanied by intimal nodular calcification in severely stenotic segments. This suggests that this predominant form represents a secondary phenomenon where eruptive intimal nodules mechanically protrude into the medial space.^5^

Conversely, only one case exhibited extensive medial sheet calcification. While this medial involvement co-existed with intimal nodules in the mid-segments, it extended prominently into distal segments lacking severe intimal narrowing (27.0% stenosis). This distinct distribution—progressing independently of severe intimal stenosis—suggests that this rare ‘true’ sheet MAC may share the pathomechanism of classic Mönckeberg-type medial sclerosis observed in peripheral vessels. In contrast, the nodular protrusion pattern represents a secondary extension of severe intimal disease rather than primary medial sclerosis. Notably, the IEL-overriding pattern occasionally seen in peripheral MAC was entirely absent in our coronary cohort.

These pathological insights have profound implications for clinical imaging. Because intimal calcified nodules cause dense acoustic shadowing or signal attenuation on intravascular imaging, any underlying medial extension (nodular protrusion) is virtually invisible *in vivo*. Furthermore, concealed elastic laminae make DIC indistinguishable from medial calcification. Therefore, the ‘coronary MAC’ frequently reported in imaging studies (7–18%) is highly unlikely to be true medial disease or nodular protrusion but rather almost exclusively represents DIC. Regarding the pathobiology of true medial disease, the rare, isolated sheet MAC in our study may share the pathomechanism of classic Mönckeberg-type medial sclerosis observed in peripheral vessels. In contrast, the nodular protrusion pattern represents a secondary extension of severe intimal disease rather than primary medial sclerosis. Notably, the IEL-overriding pattern occasionally seen in peripheral MAC was entirely absent in our coronary cohort.

These findings propose a new paradigm classifying deep coronary calcification into three distinct phenotypes: (1) DIC, the primary imaging mimic; (2) secondary nodular protrusion, which pathologically involves the media but is clinically concealed; and (3) true Mönckeberg-type medial sheet calcification, which can progress independently of severe stenosis but is exceptionally rare.

## Limitations

First, the high proportion of haemodialysis patients (21.4%) limits generalizability but was essential to capture rare MAC cases. Second, a systematic comparative analysis between histology and imaging was not performed; however, technical constraints of current imaging—such as acoustic shadowing and poor IEL visualization—preclude definitive layer-specific diagnosis *in vivo*. Finally, while specialized mineral stains were not used, Movat Pentachrome and EVG ensured the gold standard of layer-specific differentiation.

## Conclusion

True primary coronary MAC is exceptionally rare. Because acoustic shadowing conceals secondary medial nodular protrusions *in vivo*, the ‘MAC’ frequently reported in clinical imaging is almost exclusively DIC. Clinical research must account for these three distinct phenotypes—DIC, secondary nodular protrusion, and the rare true Mönckeberg-type MAC—to accurately interpret imaging findings.

## Data Availability

The data underlying this article will be shared on reasonable request to the corresponding author.
